# Advanced Characterisation of Soft Polymers under Cyclic Loading in Context of Engine Mounts

**DOI:** 10.3390/polym14030429

**Published:** 2022-01-21

**Authors:** Tomáš Gejguš, Jonas Schröder, Klara Loos, Alexander Lion, Michael Johlitz

**Affiliations:** Institute of Mechanics, Bundeswehr University Munich, 85577 Neubiberg, Germany; tomas.gejgus@unibw.de (T.G.); jonas.schroeder@unibw.de (J.S.); alexander.lion@unibw.de (A.L.); michael.johlitz@unibw.de (M.J.)

**Keywords:** engine mount, elastomer characterisation, experimental testing, resonance frequency, dynamic stiffness, parameter identification, electrodynamic shaker, test bench, cogging torque, synchronous machine

## Abstract

The experimental investigation of viscoelastic behavior of cyclically loaded elastomeric components with respect to the time and the frequency domain is critical for industrial applications. Moreover, the validation of this behavior through numerical simulations as part of the concept of virtual prototypes is equally important. Experiments, combined measurements and test setups for samples as well as for rubber-metal components are presented and evaluated with regard to their industrial application. For application in electric vehicles with relevant excitation frequencies substantially higher than by conventional drive trains, high-frequency dynamic stiffness measurements are performed up to 3000 Hz on a newly developed test bench for elastomeric samples and components. The new test bench is compared with the standard dynamic measurement method for characterization of soft polymers. A significant difference between the measured dynamic stiffness values, caused by internal resonance of the bushing, is presented. This effect has a direct impact on the acoustic behavior of the vehicle and goes undetected by conventional measurement methods due to their lower frequency range. Furthermore, for application in vehicles with internal combustion engine, where the mechanical excitation amplitudes are significantly larger than by vehicles with electric engines, a new concept for the identification of viscoelastic material parameters that is suitable for the representation of large periodic deformations under consideration of energy dissipation is described. This dissipated energy causes self-heating of the polymer and leads to the precocious aging and failure of the elastomeric component. The validation of this concept is carried out thermally and mechanically on specimen and component level. Using the approaches developed in this work, the behavior of cyclically loaded elastomeric engine mounts in different applications can be simulated to reduce the time spent and save on the costs necessary for the production of prototypes.

## 1. Introduction

The development of technically advanced vehicles or machines requires consideration of their surrounding components and of several different effects. Since electric vehicles have become established products, the focus of manufacturers is shifting to improving driving comfort. Elastomeric mounts in electric vehicles carrying motors experience loads with small amplitudes that can be applied at high frequencies. The bushings in conventional vehicles with internal combustion engines are, however, exposed to vibrations with large amplitudes in the lower frequency range. Nowadays, to decrease development costs, it is necessary to develop a concept of virtual prototypes. While the required amount of measurements decreases significantly by using virtual prototypes, some experiments are still necessary on the sample level for the identification of parameters for material models and on a component level for the validation of virtual prototypes. The material behavior of elastomeric samples or components loaded by cyclic loading is dependant on multiple factors, for instance, the static preload, frequency of the loading, dynamic amplitude, ageing condition, ambient temperature, self-heating, loading history and production instabilities. For each specific problem, with regard to the applicability and economical attractivity, different factors should be considered.

Investigations considering nonlinear viscoelasticity in the time and frequency domain can be found in Hausmann and Gergely [1], De Cazenove et al. [2], Nguyen [3] and Suphadon et al. [4], among others. Detailed descriptions of the dynamic mechanical thermal analysis (DMTA) can be found in Ehrenstein et al. [5] or Wollscheid [6]. A significant part of the noise-vibration-harshness (NVH) behavior of electric motor, called cogging, has been studied by Neapolitan and Nam [7], Hanselman [8] and Jagasics and Vajda [9]. Cogging occurs at much higher frequencies than the vibrations caused by unbalance of the rotor of synchronous machines. Lion and Johlitz [10] have shown that internal resonance of elastomeric motor mounts and cogging occur in roughly the same frequency range. Dynamic measurements in audible frequency range are presented in publications of Haeussler et al. [11], Koblar and Boltezar [12], Kari [13,14] and Ramorino et al. [15].

The characterisation of viscoelastic material behavior is the subject of ongoing research, in which Tárrago et al. [16] investigated the properties of elastomeric bearings in radial and axial direction under small amplitudes. Regarding the self-heating behavior, Behnke et al. [17] used uniaxial multi-stage tests for characterisation, whereas Kyei-Manu et al. [18] and Suphadon et al. [19] applied the cyclic stretching process with different predeformations. Mars and Fatemi [20,21] combined axial and radial cyclic loads while identifying material parameters. Carleo et al. [22] stated that, over the range of strains and strain rates that rubber-based automotive components are expected to operate in, the filled vulcanized elastomers exhibit strong nonlinearity and large hysteresis. Further possibilities for identifying parameters are, apart from the FEM, used by Gil-Negrete et al. [23], and the evaluation of measured temperature fields has been done by Balandraud et al. [24], Marco et al. [25] and Glanowski et al. [26].

First, the standardized DMTA is presented. The main advantages, possibilities and requirements of this useful measurement method are described. Additionally, measurements and the mastercurve according to time temperature shift (TTS) principle by Williams et al. [27] are shown.

For applications regarding electric vehicles, the complex NVH behavior necessitates advanced testing procedures in the frequency domain. Next, the new high-frequency test bench and its design are presented. The results from the test bench for dynamic stiffness (TBDS) are compared with the standard mastercurve. Their deviations as well as the need for such measurements for the aforementioned application are discussed along with the effects undetected by the DMTA.

Furthermore, the material characterisation approach in the time domain, with a slightly different application in vehicles with an internal combustion engine, focused on self-heating caused by dissipated energy during cyclic loading, is presented. The experimental setup is motivated, described and the measurement results are presented. The viscoelastic parameters of one characteristic hysteresis are identified through combined measurements. Finally, the validation of the identified parameter set of a component is performed and the results of the validation are discussed.

## 2. Dynamic Mechanical Thermal Analysis (DMTA)

Dynamic mechanical thermal analysis is a method to investigate dynamic material characteristics by subjecting specimens to periodic, typically sinusoidal, oscillations of small amplitude, usually under preload.

Generally, there are two ways to perform DMTA measurement:

- applying a predefined deformation and measuring the stress response
- applying a predefined stress and measuring the deformation response

**Figure 1:**
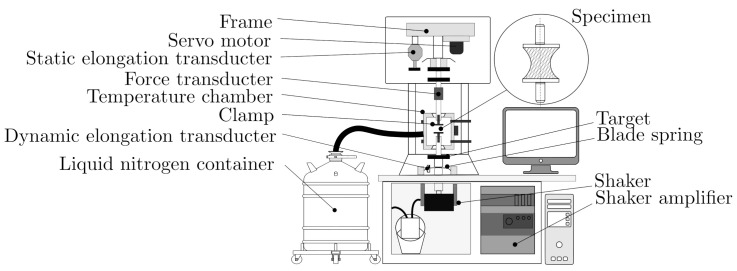
Design principle of Gabo Eplexor 500 N of DMTA testing machine.

The variety of different loading types and setups is based on the different purposes. Each different loading type is characterized by its respective advantages and difficulties. Some of the loading types require dimensionally stable samples, and others aim to investigate weak effects, thin films or anisotropic materials. Tension and compression DMTA is illustrated in Figure 1 with the design of the Gabo Eplexor 500, an experimental setup for both compression and tension testing differs only in the required clamps and sample geometry. The design consists of a static unit at the top; a dynamic unit at the bottom; and the measurement axis, including individual clamps, in the middle. The static unit is responsible for the quasi-static preload of the sample (±1500 N or ±35 mm), and its measurement enables the study of the amplitude dependence of the dynamic characteristics. The clamps are individually designed for different loading types and optimized for the precise hold of the sample. Excessive clamping can lead to measurement distortion. The dynamic unit is able to load the sample with a periodic continually controlled load (±500 N or ±1 μm up to 3 mm in range 0.01 Hz–80 Hz) and measure the dynamic response precisely. The configurable plate springs compensate for the preload and hold the coil of the electrodynamic exciter in position, so that the electrodynamic exciter is not pulled out before the dynamic load (primary voltage) is applied. The dynamic elongation is measured with an eddy current elongation sensor. Another part of the Gabo Eplexor 500 is the temperature chamber, which, in combination with liquid nitrogen, allows for measurements to be taken in a temperature range from −150 to 500 ${}^{\circ }$C.

### 2.1. Measurement Procedure

The following considerations should be taken into account when setting up the DMTA:

(**a**) DMTA experiment setup
  The DMTA testing machine is a complex device with many precise components. It is necessary to check for leaks or failures of safety components. The condition of sensors and level of liquid nitrogen are also critical factors. All these factors can lead to failure of the measurement.
(**b**) Specimen preparation, form and dimensions
  If working with dynamic moduli, it is important that the geometry of the specimen meets the requirements of technical standards and is measured precisely.
(**c**) Measurement parameters
  To start a measurement procedure, several measurement parameters have to be set. In terms of mechanical parameters, static preload, dynamic load, frequency range and division of measurement points can be defined. Within thermal parameters, the temperature range, temperature step and soak time need to be chosen. It is also necessary to set the tolerance intervals of all of the aforementioned parameters. There is no universal set of parameters to operate with when starting a new series. Obtaining a suitable set of measurement parameters is a process that consists of making several partial measurements, where every measurement point has to be logged under isothermal conditions. It is important to assure that the loaded sample is still in the so-called linear viscoelastic region (LVR), which is the main assumption that is made in DMTA measurements. In the LVR, the dynamic response (complex modulus) is not amplitude dependent. The LVR is also frequency- and temperature-dependent—lower temperatures or higher frequencies require smaller dynamic amplitudes to stay in LVR. The signal-to-noise ratio also needs to be taken into account in determination of an amplitude of dynamic load that fulfills the required conditions for the whole measurement. In general, the dynamic excitation amplitude must be small, in order for the displacement of the dynamically loaded sample to remain close to its preloaded equilibrium. Additionally, the more elastic the material is, the smaller the LVR is, and vice versa. Only if aforementioned conditions are met, it is possible to shift single frequency sweeps according to time temperature shift principle (TTS) by Williams et al. [27] and obtain a mastercurve, which represents an extrapolated dynamic response for a significantly wider frequency range than the DMTA testing machine is able to measure.

### 2.2. Dynamic Material Characteristics

After applying the load stress, the strain response can be obtained. The ratio of the dynamic stress σ to dynamic strain ε is called complex tension modulus $E^{*}$ or complex compression modulus $K^{*}$.

The real part $E^{\prime }$ ($K^{\prime }$) is the storage modulus and represents the stiffness of the viscoelastic material. It can be interpreted as energy that is stored during loading of the specimen.

The imaginary part $E^{\prime \prime }$ ($K^{\prime \prime }$), called loss modulus, represents the energy converted or dissipated as heat during mechanical loading process. This energy cannot be recovered.

In the frequency domain, the time delay between loading (dynamic strain ε) and response (dynamic stress σ) is characterized as the phase angle δ. For a purely elastic material, the phase angle δ is equal to zero.

The loss factor tanδ is defined as the ratio of the loss modulus to the storage modulus. This characteristic represents internal friction or mechanical damping; therefore, it corresponds to the amount of dissipated energy. In general, an elastic material has low loss factor.

Equations (1)–(4) describe the aforementioned relationships. Further details about dynamic material characteristics can be found in ISO-6721-1:2019 [28], ASTM-D4092:07 [29] and also in the publications by Ehrenstein et al. [5] or Meyers and Chawla [30].

(1) $$\left|E^{*}\right|=\frac{\sigma_{A}}{\varepsilon_{A}}=\sqrt{E^{\prime 2}+E^{\prime \prime 2}}$$

(2) $$E^{\prime }=\frac{\sigma_{A}}{\varepsilon_{A}}\cos\delta$$

(3) $$E^{\prime \prime }=\frac{\sigma_{A}}{\varepsilon_{A}}\sin\delta$$

(4) $$\tan\delta =\frac{E^{\prime \prime }}{E^{\prime }}$$

The glass transition temperature $T_{g}$ is one of the most important material characteristics for polymers and is defined as middle temperature of the region, in which the change from glassy or energy-elastic, to rubbery or entropy-elastic state occurs. The glass state can be interpreted as a stiff state of a polymer at low temperatures caused by immobility of its molecules. In the context of elastomers, the glass transition temperature indicates the lowest temperature at which the elastomer can be deployed for industrial application. The glass transition is also frequency-dependent.

**Figure 2:**
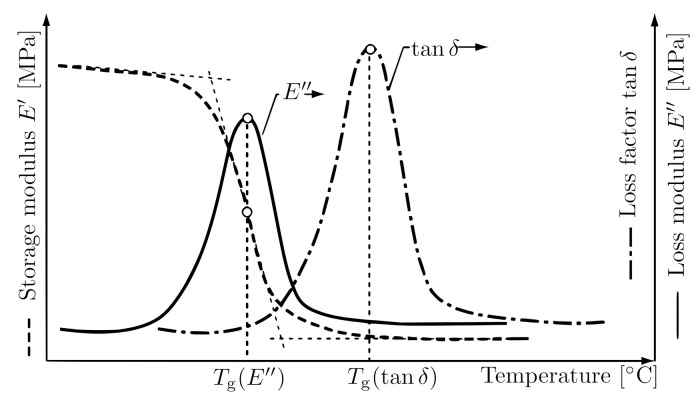
Glass transition temperature evaluation methods [5].

The value of $T_{g}$ can be determined with several methods and according to several technical norms. One commonly used method is to identify the temperature at which the maximum of loss modulus $E_{\max}^{\prime \prime }$ or maximum of loss factor ${\left(\tan\delta \right)}_{\max}$ occurs, as shown in Figure 2, as shown in Ehrenstein et al. [5]. Rieger [31] demonstrated that the $T_{g}$ obtained by the maximum of loss modulus $E_{\max}^{\prime \prime }$ coincides more with the value obtained with DSC (differential scanning calorimetry) than the value obtained with the maximum of loss factor ${\left(\tan\delta \right)}_{\max}$. The beginning of the glass transition is considered as the fall of the $E^{\prime }$.

The DMTA measurements are suitable for identification of state changes, temperature dependencies, damping dependencies, blend constituents, thermal limits of the material, anisotropy, changes due to recycling, state of ageing or conditioning, curing, and thermal degradation. For example, through a radical change in the storage modulus $E^{\prime }$, the energy- or entropy-elastic region can be identified. Different orientations of specimens of anisotropic material will each exhibit a different stiffness. The correlation between water content and change of mechanical properties can be also observed. The degree of curing of duromers can also be obtained based on the changes in moduli $E^{\prime }$, $E^{\prime \prime }$, loss factor tanδ, and rise of the glass transition temperature $T_{g}$. The thermal limits of the material can be identified by radical softening or embrittlement of the specimen. There are also many more applications for complex measurements with the DMTA, as described by Ehrenstein et al. [5].

### 2.3. Dynamic Mechanical Thermal Analysis (DMTA)—Experiment

**Figure 3:**
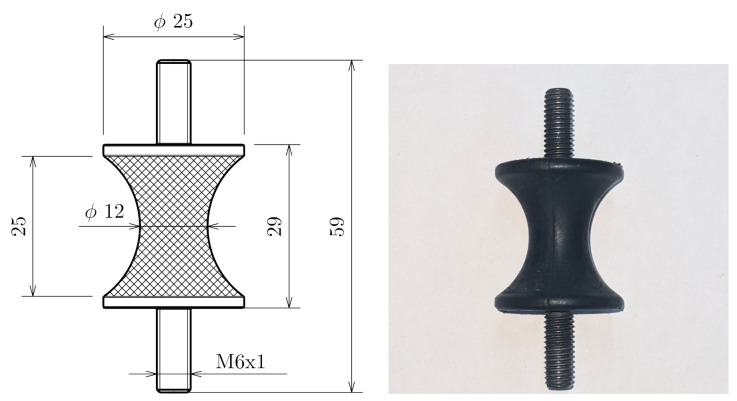
Hourglass specimen used in this study (NR-BR blend).

Figure 3 displays geometry of the specimen used in this study, along with its dimensions. The material is a carbon-black filled NR-BR (natural rubber–butadiene rubber) blend with hardness of 49 Shore A, which is typically used in rubber bushings. Since an hourglass specimen with inhomogeneous cross-section is used, the measured frequency-dependent characteristic is not the complex modulus $E^{*}$ but the dynamic stiffness $C^{*}$.

The measurement was conducted with the Gabo Eplexor 500. For this specific measurement, the 150 N force sensor and the dynamic elongation sensor with measurement range of 1.5 mm were chosen. The load parameters, such as the static elongation amplitude $\varepsilon_{{\mathrm{st}}_{A}}=0.5$% and the dynamic elongation amplitude $\varepsilon_{{\mathrm{dyn}}_{A}}=0.2$%, were set up carefully under consideration of the LVR and the initial length of the specimen $L_{0}$ = 25 mm. The temperature range between −40 ${}^{\circ }$C and 20 ${}^{\circ }$C was used for the temperature sweep of individual isothermal frequency sweeps, which were measured from 1–80 Hz with logarithmic distribution of measurement points 12 Points/decade.

**Figure 4:**
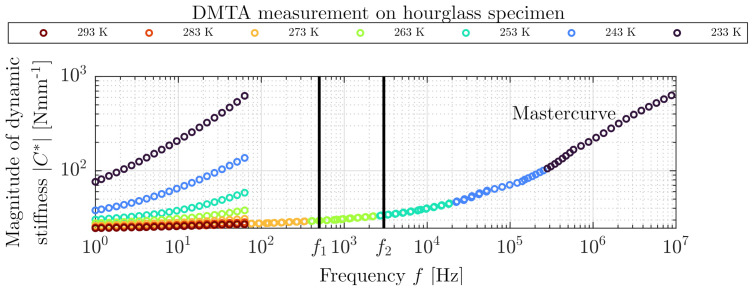
DMTA measurement in frequency range of 1–80 Hz and mastercurve for 1–$10^{7}$ Hz of carbon-black filled elastomeric hourglass specimen.

Figure 4 shows the magnitude of the dynamic stiffness $\left|C^{*}\right|$ of the hourglass elastomeric specimen, which was obtained by a temperature-frequency sweep with the DMTA. The corresponding mastercurve was extrapolated by TTS according to Williams et al. [27]. The first vertical line $f_{1}$ indicates the frequency range 0–500 Hz. The second line $f_{2}$ indicates the frequency range 0–3000 Hz, which will be used for measurements in Section 3.

## 3. High-Frequency Dynamic Testing (Frequency Domain)

There is a new sector of testing machines, which are able to test samples and components at high frequency, loaded by sinusoidal load, at room temperature and non-destructively. This sector of test machines is motivated by NVH behaviour of electric vehicles because the design and working principle of an electric motor is different from an internal combustion engine.

**Figure 5:**
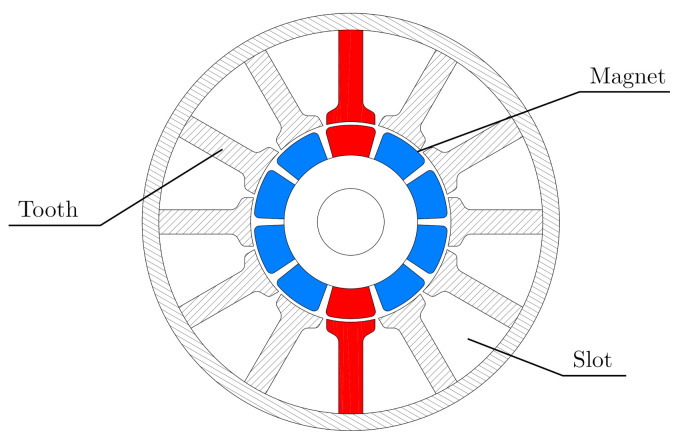
Permanent magnet synchronous motor (PMSM)—cross section (10 Poles, 12 Coils).

The rotational frequency of a standard internal combustion engine can reach up to 100 Hz. As a result, the relevant frequency region typically used in industry measurements is from 0 to 500 Hz. Permanent magnet synchronous motors (PMSM) of an electric motor can, however, operate with a rotational frequency of up to 300 Hz. Due to the working principle of the PMSM, further superimposed effects can be found in its NVH behavior. In any spectrum of electronic commutated or brushless motors, the pulse width modulation (PWM) effect occurs with the frequency of the motor controller. The cogging torque occurs in every permanent magnet machine and is defined as the torque ripple, which is caused by the uneven attraction of the magnets on the rotor to the teeth and slots on the stator. In the position where the pole aligns with the teeth, the highest attraction or the maximum of magnetic flux density occurs—shown schematically in Figure 5. Neapolitan and Nam [7] state that the cogging torque depends on the rotational frequency, on the number of poles and slots and on the load of the motor. There are several strategies to compensate for this undesired effect. The magnets can be segmented and arranged in several ways or the coils can be switched smoothly, so that they act against the cogging torque, according to Hanselman [8]. The cogging frequency is linked to the least common multiple of the number of poles and slots, as observed by Jagasics and Vajda [9]. Since the vibrations generated by a PMSM occur often at high frequencies, it is necessary to include higher frequencies in the testing standards. These high-frequency effects cause internal resonances of the rubber bushings during the operation of permanent magnet machines, as demonstrated by Lion and Johlitz [10].

### 3.1. Test Bench for Dynamic Stiffness (TBDS)—Design

There are several scientific publications which deal with high-frequency dynamic testing of elastomers, using different designs of the test apparatus, as can be seen in Ramorino et al. [15] and Koblar and Boltezar [12]. Haeussler et al. [11] performed “free–free” rubber isolator measurements to be able to describe the vibration in virtual points and compute the frequency dependent dynamic stiffness. Testing machines to measure the dynamic stiffness of elastomers within high frequencies are also commercially available.

**Figure 6:**
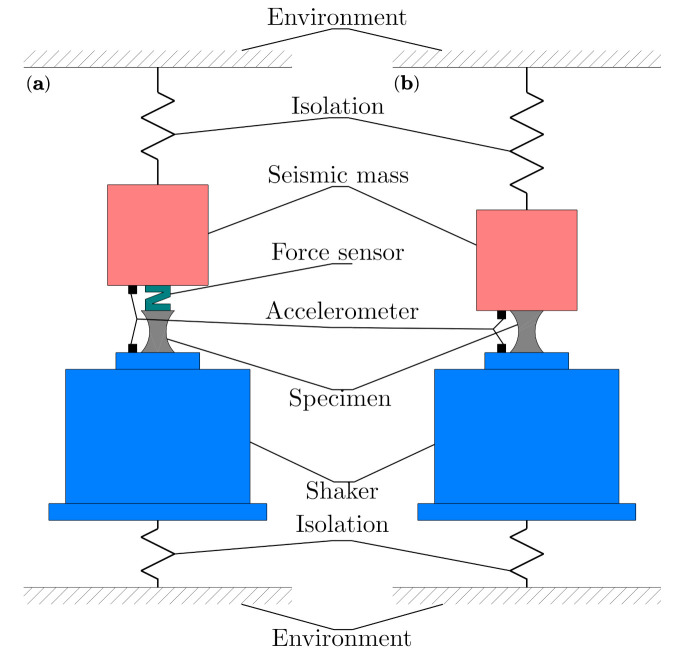
Design concept of test bench for measurement of dynamic stiffness: (**a**) direct method, (**b**) indirect method.

In addition to the approach of Haeussler et al. [11], there are two possibilities for measuring dynamic characteristics of rubber bushing in a wide frequency range, as outlined in Figure 6. The measurement method can be performed, as shown in Figure 6a, directly, utilizing an accelerometer and force sensor, or indirectly, using two accelerometers as in Figure 6b. The disadvantage of the direct method is its more complex design and generally lower resonance frequency of the force sensors.

**Figure 7:**
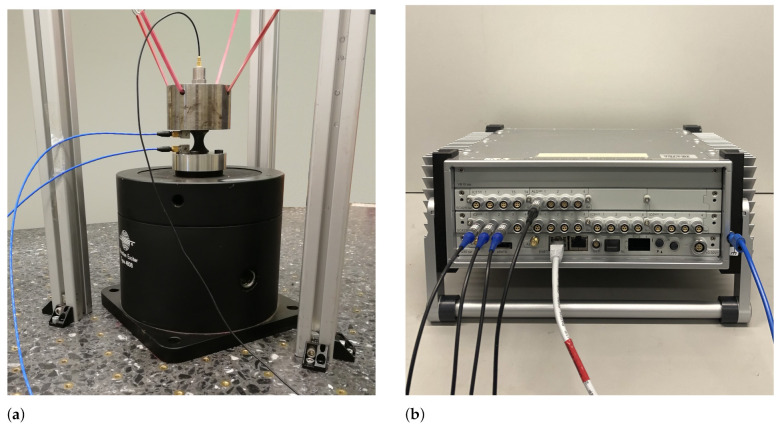
Experiment: (**a**) Test bench for dynamic stiffness (TBDS), (**b**) multichannel measurement system. PAK MKII by Müller BBM VAS.

In the current study, the indirect method was chosen and manufactured on a 500 kg stone table with thread inserts isolated by rubber air springs, as shown in Figure 7a. It is important that the whole apparatus remains isolated from the environment. The seismic mass was hung “free-free” and parallel to the table of dynamic exciter. The solid body resonance of the seismic mass attached with the elastic support cords was outside the measurement range, so that sinusoidal dynamic load could be applied. In this case, the electrodynamic exciter B&K Type 4808 with maximum table acceleration of 71 g powered by B&K Type 2712 was used. This exciter delivers up to 187 N peak sine force in range from 5 Hz to 10 kHz. The accelerometers from PCB with sensitivity of 10.29 ${\mathrm{mVg}}^{-1}$ and B&K with sensitivity of 98.5 ${\mathrm{pCg}}^{-1}$ were utilized. It is preferred that the cables are fixed and have no loops. The measurements were performed on the modular multichannel measurement system PAK MKII by Müller BBM VAS (see Figure 7b) using the corresponding software PAK 6.0, and the excitation velocity amplitude was set to $v\;=\;0.01\;{\mathrm{ms}}^{-1}$ with tolerance of 5% during the entire measurement from 50–3000 Hz with a frequency step of 1 Hz. The set velocity amplitude was controlled by real-time closed loop offered by PAK 6.0. The sampling rate was set up with respect to the Nyquist criterion. Three analog inputs and one analog output were used to conduct the measurement. The direct computation of dynamic stiffness of the bushing was carried out using the Arithmetic toolbox in PAK 6.0. The measurement setup in this study is named “Test bench for dynamic stiffness” (TBDS). The TBDS is able to test different specimens and components made from soft or hard polymers since the mass of adaption can be easily compensated. For hard polymers, the dynamic amplitude has to be reduced regarding the power of the dynamic exciter in order to reach 3000 Hz. Concurrently, it is important to consider the measurement noise of the used accelerometers.

### 3.2. Test Bench for Dynamic Stiffness (TBDS)—Experiment

**Figure 8:**
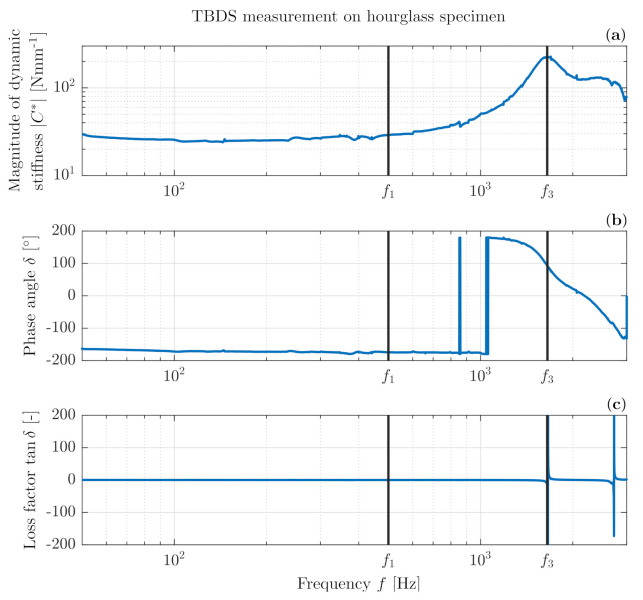
Measurement on hourglass specimen from TBDS.

Figure 8 shows a successful isothermal frequency sweep measurement from the TBDS over a large frequency range of 50–3000 Hz of the same hourglass specimen as used in the DMTA temperature frequency sweep in Section 3. The magnitude of dynamic stiffness $\left|C^{*}\right|$ (Figure 8a), phase angle δ (Figure 8b) and loss factor tanδ (Figure 8c) are shown as a factor of frequency. The thick blue vertical lines in Figure 8b at close proximity of $10^{3}$ Hz are caused by measurement noise and plotting method (−180${}^{\circ }$ up to 180${}^{\circ }$) of phase angle δ. The first vertical line $f_{1}$ marks 500 Hz, which is the region where industrial measurements usually take place. Clearly, the dynamic stiffness can be considered as constant from 50 to 500 Hz. The second vertical line $f_{3}$ marks the maximum of the magnitude of dynamic stiffness $\left|C^{*}\right|$ at approx. 1650 Hz, which indicates an internal resonance of the test specimen. This is confirmed by the phase angle δ, which is approximately 90${}^{\circ }$ at this resonance frequency.

### 3.3. Test Bench for Dynamic Stiffness (TBDS)—Validation (DMTA)

**Figure 9:**
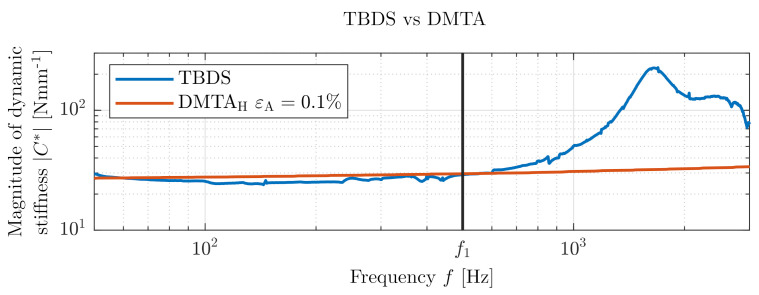
Comparison—TBDS vs. DMTA (hourglass specimen).

In Figure 9, the magnitude of the dynamic stiffness $\left|C^{*}\right|$ measured on our test bench is plotted in comparison with the mastercurve shifted from temperature frequency sweep performed on DMTA with the same specimen, as shown in Figure 4. In the low-frequency range up to 500 Hz marked with line $f_{1}$, which is usually used for industrial measurements of rubber bushings, both measurements correlate well with each other. At higher frequencies, a prominent increase of the magnitude of dynamic stiffness $\left|C^{*}\right|$ was measured by TBDS. The first internal resonance of the rubber bushing occurs near 1650 Hz. The standard temperature frequency sweep (DMTA measurement) proceeded up to 100 Hz and then shifted according to TTS (a mastercurve can never yield any information about internal resonance that occurs outside the frequency range of the frequency sweep). With the mastercurve from the DMTA measurements, the significant increase of dynamic stiffness remains undetected. The DMTA is a versatile useful standard analysis; nevertheless, there is a need, caused by NVH behavior of PMSM, to measure dynamic stiffness at high frequencies for applications in electromobility. Table 1 shows clearly arranged comparison of pros and cons of the TBDS and the DMTA.

## 4. Dissipative Self-Heating of Engine Mounts (Time Domain)

**Figure 10:**
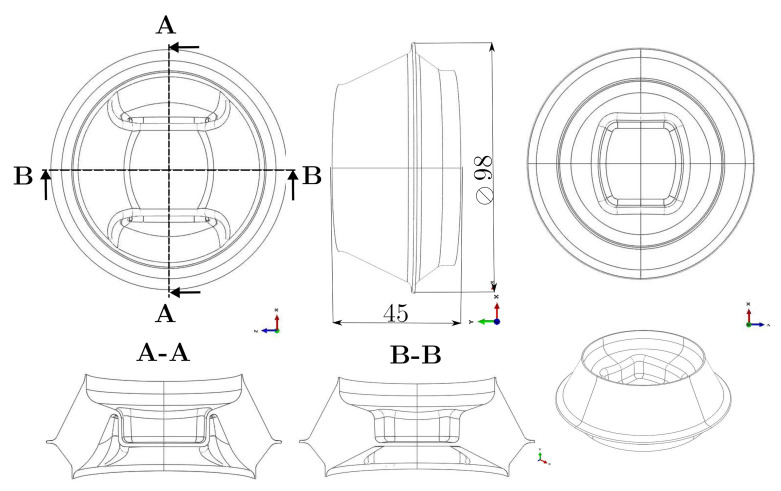
Engine mount for vehicles with internal combustion engines.

In contrast to electric machines, bearings used for internal combustion engines (Figure 10) are subjected to tendentially lower frequencies combined with relatively large amplitudes. Cyclic mechanical loads at sufficiently high amplitudes and frequencies lead to significant energy dissipation in viscoelastic materials. In the case of insufficient heat removal, elastomer components may therefore heat up strongly as shown, for example, by Johlitz et al. [32] or Dippel et al. [33]. The occurrence of critical temperatures, which can lead to loss of functionality or to component failure, has to be identified at an early stage in the development process. For reasons of time and cost, simulation is now replacing a large part of the experimental work. However, in addition to suitable material models, this also requires suitable methods and equipment for material characterisation, as the publication by Hartmann [34] describes. This section, therefore, focuses on the viscoelastic material characterisation, which, in the context of dissipative self-heating, has a significant influence on the valency, progress and stability of the resulting temperature field, which is examined in more detail within the study published by Schröder et al. [35]. The present study presents a method for experimental identification of viscoelastic parameters, which can be transferred to complex geometries and multi-axial deformation states and velocities. An overall estimation is made concerning the industrial applicability, which includes not only the duration and quality of the tests but also the total experimental effort for the identification of the material parameters.

### 4.1. Experimental Setup and Time Domain Procedures

Classical methods such as the relaxation test or the dynamic mechanical analysis do not represent the required relaxation spectrum in the time or frequency range if an amplitude spectrum within a frequency band has to be described. This issue is also addressed by Lion [36] and further investigated experimentally by Dippel et al. [33], among others. Furthermore, a large number of technical elastomer materials show pronounced non-linear viscoelastic material behavior, which is generally not simulated for reasons of efficiency but which must be taken into account during parameter identification. This was investigated, for instance, in the work of Koprowski-Theiß [37] and Scheffer [38], as well as Schröder et al. [39] and many other authors. In the following, the experimental method and the experimental setup are described. The ElectroForce 3200 Series III DMA testing machine from BOSE is used for mechanical testing within the ETI-2 temperature chamber. The following tests are conducted using carbon black-filled natural rubber (NR) samples cross-linked by low-sulphur vulcanisation (Efficient Vulcanisation, EV). Further parameters of the material are the SHORE hardness of 68 ShA, an Elongation at Break of 366%, and a Rebound Resilience of 31%. Periodic uniaxial tensile tests were carried out on simple rectangular specimens 10 mm × 1.8 mm × 50 mm until a stationary temperature field was reached. The process was characterized by a swelling displacement process and varied in terms of frequencies 1, 2, 5 and 10 Hz and strain amplitudes 5, 10 and 15%. The field quantities force and displacement were recorded by sensors. In parallel, the evolution of the surface temperature of the sample was captured by an infrared camera of the type VarioCam and the ambient temperature by a thermal sensor. These measured data provided the basis for the parameter identification, and they were used for the later validation of the parameter set.

### 4.2. Parameter Identification Using Modified Ellipse Function

**Figure 11:**
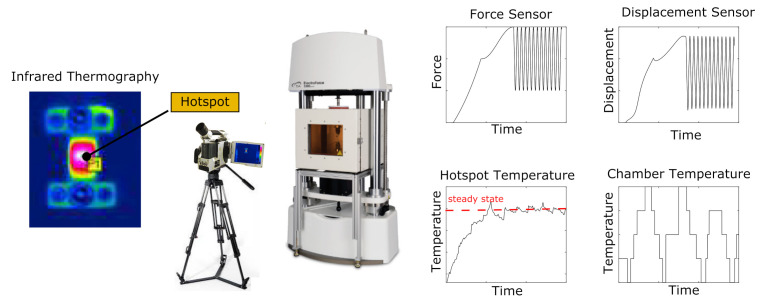
Experimental setup and data acquisition.

**Figure 12:**
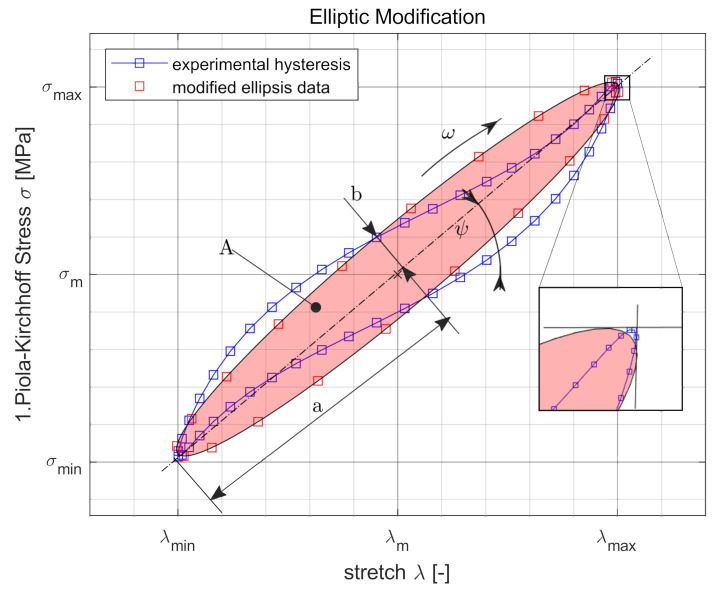
Edited measurements in a hysteresis representation and an elliptical approximation curve.

The measured data are edited and formatted. The surface temperature evaluated at the location ‘Hotspot’ where the maximum temperature occurs and the mean value of the oscillating temperature of the last cycles is used as the steady-state equilibrium temperature. This is shown in Figure 11 and summarized in the following as a function of the input process variables. The mechanical measurement data are converted into stretch λ and stress σ by using the geometry of the specimen. In addition to the stationary temperature of the process, a representative cycle is selected and the relevant characteristic properties such as extremal values $\left(\lambda_{min}/\sigma_{min}\right)$ and $\left(\lambda_{max}/\sigma_{max}\right)$ and the area *A* of the associated hysteresis are calculated. Subsequently, this is determined as in Figure 12 and is replaced by an ellipse function $e\left(\sigma_{m},\lambda_{m},a,b,\psi ,f,t\right)$ depending on the parameters for transformation $\sigma_{m}$ and $\lambda_{m}$, rotation ψ, geometry *a* and *b*, frequency *f* and time *t*.

(5) $$\begin{array}{c} \left[\begin{array}{c} \lambda \\ \sigma \end{array}\right]=\left[\begin{array}{c} \lambda_{m}\\ \sigma_{m} \end{array}\right]+\frac{1}{2}\;a\;\cos\left(2\;\pi \;f\;t\right)\;\left[\begin{array}{c} \cos\left(\psi \right)\\ \sin\left(\psi \right) \end{array}\right]+2\;b\;\sin\left(2\;\pi \;f\;t\right)\;\left[\begin{array}{c} -\sin\left(\psi \right)\\ \cos\left(\psi \right) \end{array}\right] \end{array}$$

The target function $z\left(p\left(\eta_{i}\;\mu_{i}\right)\right)$ is defined on the basis of the abstracted data of the measurement $T^{\mathrm{abs}}\left(p\right)$ and the process response of the material model $T^{\mathrm{mod}}\left(p\right)$, with the viscoelastic model parameters p, viscosities $\eta_{i}$ and stiffnesses $\mu_{j}$ being used as variables. To solve the inverse problem, an error function is first introduced, which represents a quality criterion between simulation and experiment.

(6) $$z\left(p\left(\eta_{i},\mu_{i}\right)\right)=∥\sum_{j}T_{j}^{\mathrm{mod}}\left(p\right)-T_{j}^{\mathrm{abs}}∥\to \;\mathrm{minimal}$$

**Figure 13:**
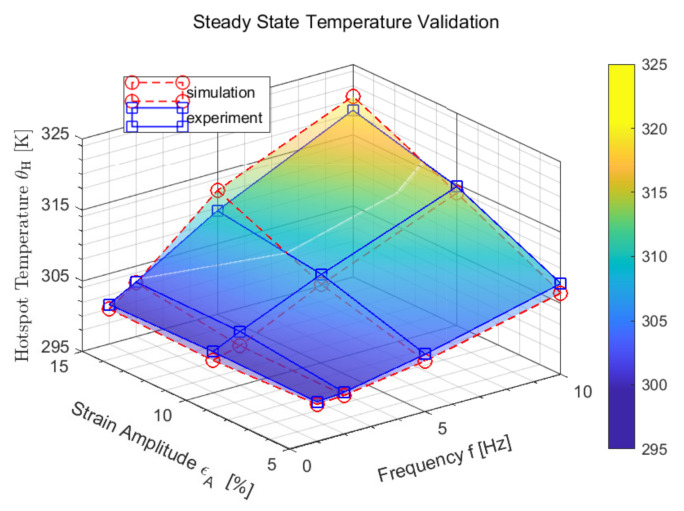
Experimental and simulative steady-state temperatures under not released swell strain conditions.

To optimize the target function, a genetic algorithm based on the mutation-selection principle and the recombination mechanism as well as a gradient-based method are chosen and combined through iteration loops, as Koprowski-Theiß [37] presents in her work. In this manner, advantages of the evolution strategy and the start and extreme value finding can be used to find the best available set of parameters. A numerical simulation is used to replicate the experiment and to validate it at sample level using the determined set of parameters. The extremely satisfactory agreement of the results is shown in Figure 13.

### 4.3. Parameter Validation at Component Level

**Figure 14:**
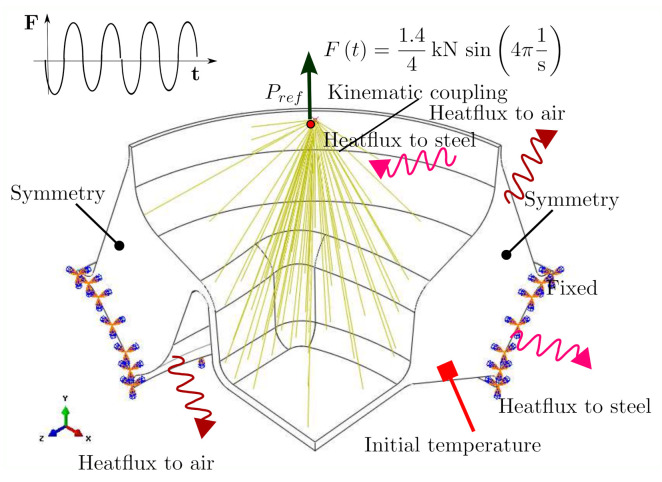
Computational model showing boundary and initial conditions.

The formulation of the initial and boundary conditions shown in Figure 14 is mandatory for the model within a fully coupled thermomechanical analysis. The analysis is transient with respect to the heat conduction equation, whereas inertia effects are neglected. The heat transfer equation is integrated using a backward-difference scheme. The incrementation is done automatically under the restriction of used increment sizes between $5\cdot 10^{-5}\;\left[s\right]$ and $5\cdot 10^{-2}\;\left[s\right]$.

**Figure 15:**
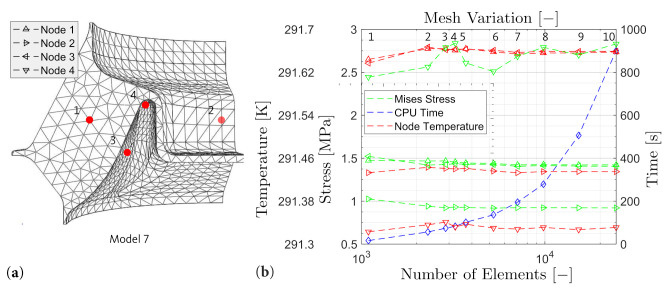
(**a**) Definition of reference nodes and Model mesh. (**b**) Convergence study.

A qualitatively valuable discretisation conserves computational resources while ensuring the accuracy of the results. For this purpose, convergence and mesh independence must be proven. To fulfill these requirements, a mesh convergence analysis is carried out. A representative load case is evaluated where the number of elements is varied. The temperature and Mises stress as well as the total computation time for each mesh (model 1–model 10) are determined at four characteristic nodes (Node 1–Node 4). The different discretisations and evaluated locations are shown in Figure 15a.

With the use of the convergence, the element size or number can be determined where the results do not significantly change any longer. At this point, mesh variation 7, the model is independent of the mesh and leads neither to increased computation times nor to discretisation erros as shown in Figure 15b. The geometry is discretized by 7093 C3D10MHT (three-dimensional 10-node modified temperature-displacement hybrid tetrahedron element with linear pressure and with hourglass control) elements.

**Figure 16:**
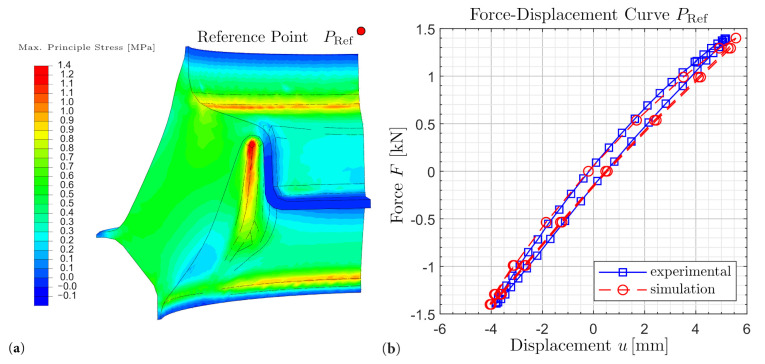
(**a**) ABAQUS analysis stress visualisation engine mount. (**b**) Force-displacement curve at the reference point.

**Figure 17:**
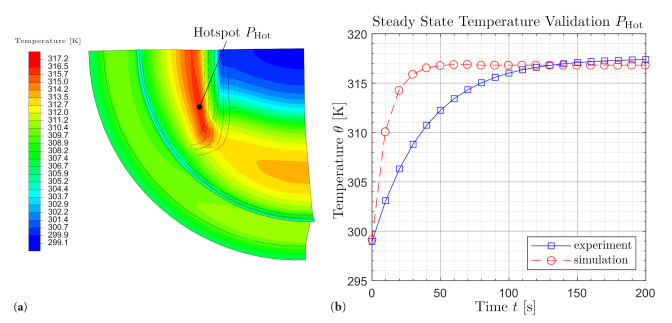
(**a**) ABAQUS analysis temperature visualisation engine mount. (**b**) Self-heating under cyclic loading.

The identified parameter set is now used to compute the mechanical and thermal behavior of an engine mount as it is subject to complex load and deformation conditions. These are multi-axial as well as location- and time-dependent. The calculation results are presented in Figure 16b and Figure 17b. For modelling, implementation and optimization of the computation time refer to Schröder et al. [39,40].

In Figure 16b, a nearly identical hysteresis is observed. This means that in addition to the mechanical behavior, the dissipative behavior, which is characterized by the hysteresis area, is also captured. Accordingly, the local loads can be deduced at this point. The agreement of the stationary temperature values between experiment and simulation can be recognized in Figure 17b. It is also observed that the calculation duration is significantly reduced by the suitable selection of the heat capacity. For detailed descriptions and proof of validity, refer to Schröder et al. [40]. The reach of the stationary state is thereby fastened. Furthermore, the local temperature profile in the stationary state can be inferred, as shown in Figure 17b.

## 5. Summary and Outlook

It is important to verify established procedures since the possibilities and problems are changing. The described DMTA analysis is a useful multifunctional measurement method; however, for several applications, it is necessary to consider effects that remain undetected by this or any other standard method.

For applications in electric vehicles, the vibrations caused by PMSM are of small amplitude with wide frequency range. The characteristic NVH behavior of PMSM is caused by the superposition of multiple mechanical and electromagnetic effects. It was shown on measurements from our beta version of the test bench for measurement of dynamic stiffness and DMTA that the standard measurement procedure has its limitations. The comparison of the two mentioned measurements was discussed, and the reason for the significant difference was stated. The TBDS measurements can be performed on specimens to obtain parameters for material model of frequency dependent moduli and also for validation of simulations of the virtual prototype, where dynamic load is applied on the elastomer bushing. With the help of such models, it is possible to simulate dynamic response of the rubber isolator; compare it with spectrum of the PMSM; and, if it is needed, change the design or position of the isolator to avoid undesired NVH behavior before the start of the production. It is also possible to test aged specimens to obtain information about the change of the spectrum of rubber isolator caused by ageing. In the future, the design will be improved to be able to set up the preload precisely and some components will be replaced by higher quality components. Eventually, the model according to Lion and Johlitz [10] will be fitted and validated on measurements from TBDS.

The second application in this study is adapted on NVH behavior of internal combustion engines. The cyclic, low frequency load, high amplitudes and long operation times are affecting the elastomer bushing. It was shown on measurements that the energy dissipated by loading of elastomer specimen causes its self-heating. At this point, the standard DMTA reaches its limits as well. In this study, the combined mechanical and thermal measurement for the quantification of dissipative heating of elastomer specimen is described and the steady state temperatures are obtained. The results of previously mentioned measurements are used for parameter identification of the hysteresis area for the computation of the characteristic ellipse. The identified parameter set is used for simulation on the component level and validated with respect to combined measurement of engine mount. The effect of dissipative self-heating was characterized by a minimal number of experiments at the specimen level, and the developed model is suitable for transfer to the complex geometry and load cases of the component (engine mount).

With the help of these approaches, the behavior of cyclic loaded elastomer bushing in different applications can be simulated to save the time and costs necessary for the production of prototypes or losses caused by possible complaints from customers.

## Figures and Tables

**Table 1:** Pros and cons of TBDS and DMTA.

	TBDS	DMTA
Pros	wide frequency range	controlled preload
	detection of internal resonance	temperature control
	controlled dynamic load	controlled dynamic load
	open parameters and low costs	user-friendly and established
Cons	constant preload	small frequency range
	no temperature control	component testing difficulties
	user comfort	high acquisition costs

## Data Availability

Not applicable.

## References

[1] Hausmann G., Gergely P. Approximate methods for thermoviscoelastic characterization and analysis of elastomeric lead-lag dampers. Proceedings of the AAAF, 18th European Rotorcraft Forum.

[2] De Cazenove J., Rade D., De Lima A., Araújo C. (2011). A numerical and experimental investigation on self-heating effects in viscoelastic dampers. Mech. Syst. Signal Process..

[3] Nguyen N.T. (2014). Experiments and Inverse Analysis for Determining Non-Linear Viscoelastic Properties of Polymeric Capsules and Biological Cells. Ph.D. Thesis.

[4] Suphadon N., Thomas A., Busfield J. (2010). The viscoelastic behavior of rubber under a complex loading. II. The effect large strains and the incorporation of carbon black. J. Appl. Polym. Sci..

[5] Ehrenstein G., Riedel G., Trawiel P. (2012). Thermal Analysis of Plastics: Theory and Practice.

[6] Wollscheid D. (2014). Predeformation- and Frequency-Dependent Material Behavior of Filler-Reinforced Rubber: Experiments, Constitutive Modelling and Parameter Identification. Ph.D. Thesis.

[7] Neapolitan R.E., Nam K.H. (2018). AC Motor Control and Electrical Vehicle Applications.

[8] Hanselman D.C. (2003). Brushless Permanent Magnet Motor Design.

[9] Jagasics S., Vajda I. (2016). Cogging torque reduction by magnet pole pairing technique. Acta Polytech. Hung..

[10] Lion A., Johlitz M. (2020). A mechanical model to describe the vibroacoustic behavior of elastomeric engine mounts for electric vehicles. Mech. Syst. Signal Process..

[11] Haeussler M., Klaassen S., Rixen D. (2020). Experimental twelve degree of freedom rubber isolator models for use in substructuring assemblies. J. Sound Vib..

[12] Koblar D., Boltezar M. (2013). Evaluation of the Frequency-Dependent Young’s Modulus and Damping Factor of Rubber from Experiment and Their Implementation in a Finite-Element Analysis. Exp. Tech..

[13] Kari L. (2001). Dynamic transfer stiffness measurements of vibration isolators in the audible frequency range. Noise Control. Eng. J. Noise Control Eng..

[14] Kari L. (2003). On the dynamic stiffness of preloaded vibration isolators in the audible frequency range: Modeling and experiments. J. Acoust. Soc. Am..

[15] Ramorino G., Vetturi D., Cambiaghi D., Pegoretti A., Ricco T. (2003). Developments in Dynamic Testing of Rubber Compounds: Assessment of Non-Linear Effects. Polym. Test..

[16] Tárrago M.G., Kari L., Vinolas J., Gil-Negrete N. (2007). Frequency and amplitude dependence of the axial and radial stiffness of carbon-black filled rubber bushings. Polym. Test..

[17] Behnke R., Kaliske M., Klüppel M. (2016). Thermo-mechanical analysis of cyclically loaded particle-reinforced elastomer components: Experiment and finite element simulation. Rubber Chem. Technol..

[18] Kyei-Manu W.A., Tunnicliffe L.B., Plagge J., Herd C.R., Akutagawa K., Pugno N.M., Busfield J.J. (2021). Thermomechanical Characterization of Carbon Black Reinforced Rubbers During Rapid Adiabatic Straining. Front. Mater..

[19] Suphadon N., Thomas A., Busfield J. (2009). Viscoelastic behavior of rubber under a complex loading. J. Appl. Polym. Sci..

[20] Mars W., Fatemi A. (2004). A novel specimen for investigating the mechanical behavior of elastomers under multiaxial loading conditions. Exp. Mech..

[21] Mars W., Fatemi A. (2004). Factors that affect the fatigue life of rubber: A literature survey. Rubber Chem. Technol..

[22] Carleo F., Barbieri E., Whear R., Busfield J.J. (2018). Limitations of viscoelastic constitutive models for carbon-black reinforced rubber in medium dynamic strains and medium strain rates. Polymers.

[23] Gil-Negrete N., Vinolas J., Kari L. (2006). A simplified methodology to predict the dynamic stiffness of carbon-black filled rubber isolators using a finite element code. J. Sound Vib..

[24] Balandraud X., Toussaint E., Le Cam J., Grédiac M., Behnke R., Kaliske M. (2011). Application of full-field measurements and numerical simulations to analyze the thermo-mechanical response of a three-branch rubber specimen. Const. Model. Rubber VII.

[25] Marco Y., Le Saux V., Jégou L., Launay A., Serrano L., Raoult I., Calloch S. (2014). Dissipation analysis in SFRP structural samples: Thermomechanical analysis and comparison to numerical simulations. Int. J. Fatigue.

[26] Glanowski T., Le Saux V., Doudard C., Marco Y., Champy C., Charrier P. (2017). Proposition of an uncoupled approach for the identification of cyclic heat sources from temperature fields in the presence of large strains. Contin. Mech. Thermodyn..

[27] Williams M.L., Landel R.F., Ferry J.D. (1955). The Temperature Dependence of Relaxation Mechanisms in Amorphous Polymers and Other Glass-forming Liquids. J. Am. Chem. Soc..

[28] ISO-6721-1:2019 (2019). Plastics—Determination of Dynamic Mechanical Properties—Part 1: General Principles.

[29] ASTM-D4092:07(2013) (2013). Standard Terminology: Plastics: Dynamic Mechanical Properties.

[30] Meyers M.A., Chawla K.K. (2009). Mechanical Behavior of Materials.

[31] Rieger J. (2001). The glass transition temperature Tg of polymers-Comparison of the values from differential thermal analysis (DTA, DSC) and dynamic mechanical measurements (torsion pendulum). Polym. Test..

[32] Johlitz M., Dippel B., Lion A. (2016). Dissipative heating of elastomers: A new modelling approach based on finite and coupled thermomechanics. Contin. Mech. Thermodyn..

[33] Dippel B., Johlitz M., Lion A. (2015). Thermo-mechanical couplings in elastomers–experiments and modelling. ZAMM-J. Appl. Math. Mech./Z. Angew. Math. Mech..

[34] Hartmann S. (2019). From Experiments to Predicting the Component Behavior in Solid Mechanics.

[35] Schröder J., Lion A., Johlitz M. (2021). Numerical studies on the self-heating phenomenon of elastomers based on finite thermoviscoelasticity. J. Rubber Res..

[36] Lion A. (2000). Thermomechanik von Elastomeren.

[37] Koprowski-Theiß N. (2011). Kompressible, Viskoelastische Werkstoffe: Experimente, Modellierung und FE-Umsetzung. Ph.D. Thesis.

[38] Scheffer T. (2016). Charakterisierung des Nichtlinear-Viskoelastischen Materialverhaltens Gefüllter Elastomere. Ph.D. Thesis.

[39] Schröder J., Lion A., Johlitz M. (2019). On the Derivation and Application of a Finite Strain Thermo-viscoelastic Material Model for Rubber Components. State of the Art and Future Trends in Material Modeling.

[40] Schröder J., Lion A., Johlitz M. (2019). Thermoviscoelastic modelling of elastomer components in industrial applications. Constitutive Models for Rubber XI, Proceedings of the 11th European Conference on Constitutive Models for Rubber (ECCMR 2019), Nantes, France, 25–27 June 2019.

